# Gastric Myoelectric Activity and Body Composition in Women with Binge Eating Disorder and Bulimia Nervosa: A Preliminary Trial Study

**DOI:** 10.3390/jcm12144563

**Published:** 2023-07-08

**Authors:** Seham H. Alyami, Adel Alhamdan, Hanan M. Alebrahim, Ahmad H. Almadani, Ghadeer S. Aljuraiban, Mahmoud M. A. Abulmeaty

**Affiliations:** 1 Community Health Sciences Department, College of Applied Medical Sciences, King Saud University, Riyadh 11433, Saudi Arabia; 442204326@student.ksu.edu.sa (S.H.A.); adel@ksu.edu.sa (A.A.); galjuraiban@ksu.edu.sa (G.S.A.); 2 Department of Psychiatry, College of Medicine, King Saud University, Riyadh 12372, Saudi Arabia; haalebrahim@ksu.edu.sa (H.M.A.); ahalmadani@ksu.edu.sa (A.H.A.)

**Keywords:** eating disorders, binge eating disorder, bulimia nervosa, electrogastrography, gastric myoelectric activity

## Abstract

Women with eating disorders (EDs) often complain of abnormal gastric responses, which may impact their eating patterns and, consequently, their body composition. Binge eating disorder (BED) and bulimia nervosa (BN) have been shown to affect gastric myoelectric activity (GMA), which may provide a basis for the gastric response in this disease population. This study aimed to examine GMA and body composition in patients with an ED compared to age—body mass index (BMI) matched controls. This case—control study included 18 adults diagnosed with BED or BN compared to 19 age—gender-BMI-matched controls. The electrogastrography with water load test was used to measure GMA during fasting and after water loading to satiety. Body composition was measured using a bioelectric impedance analyzer. The results showed that the ED group had a significantly higher water load than the control group and increased percentages of tachygastria times. Comparing the BED and BN subgroups showed differences in body composition status between the subgroups in the form of less fat mass, muscle mass, and total body water in the BN subgroup. In the BN subgroup, fat mass was associated with the average dominant frequency in the EGG. Thus, measuring GMA may be a promising approach to understanding gastric abnormalities in patients with EDs. Therapies targeting improving body composition in women with BED and BN are recommended in future ED management strategies.

## 1. Introduction

Binge eating disorder (BED) and bulimia nervosa (BN) are eating disorders (EDs) characterized by episodes of binge eating, that is, eating a large amount of food with a loss of control, in a short period. BN is also characterized by compensatory behaviors, such as self-induced vomiting, fasting, compulsive exercise, and laxative or diuretic use, whereas in BED, regular compensatory behaviors are absent [1]. Both were added to the Diagnostic and Statistical Manual of Mental Disorders, 5th edition (DSM-5) [2]. The most recent worldwide prevalence of BED in adults was 0.6–1.8% in women and 0.3–0.7% in men [3]. Based on the Saudi National Mental Health Survey in the Kingdom of Saudi Arabia (KSA), the lifetime prevalence in 2019 was 3.3% and 2.9% for binge eating disorder (BED) and bulimia nervosa (BN), respectively [4]. Early diagnosis and management of EDs and the associated medical comorbidities may improve the response and clinical outcomes [5]. 

Consequently, individuals with BED are usually suffering from being overweight or obese [6], with associated several comorbidities, such as type 2 diabetes, hypertension, dyslipidemia, sleep disorders, and gastrointestinal symptoms/disorders [7]. Moreover, body composition changes in BED and BN are interesting points of research. Mathisen et al. [8] reported higher body mass index (BMI), visceral adipose tissue (VAT), and percent body fat in females with bulimia nervosa or binge eating disorder. A recent systematic review and meta-analysis explored the interactions between the body composition of patients with EDs and hormonal changes and low bone mineral density. They recommended the repeated use of non-invasive and easy-to-operate methods such as Bioelectrical Impedance Analysis (BIA) to track body composition changes regarding fat and fat-free mass before, during, and after ED treatment [9]. 

The pathophysiology of BED and BN is not well understood [10]. The etiology of ED is multifactorial and may involve genetic factors, an altered food reward system, low self-esteem, dieting, or a history of dieting [11,12]. Identifying the factors affecting food intake and contributing to binge eating will facilitate proper intervention and treatment. Furthermore, individuals with eating disorders often complain of gastrointestinal issues, such as nausea, bloating, delayed gastric emptying, constipation, or diarrhea [13], which have been associated with gastric dysrhythmias (tachygastria and/or bradygastria) [14]. Rebollo et al. [15] found that the functional MRI studies showed that the brain is coupled to the gastric rhythm in many cortical pathways, including the primary and secondary somatosensory cortices and the parieto-occipital region. Gastric–brain coupling and the study of brain–viscera interactions are hot areas of research.

Electrogastrography (EGG) is a non-invasive procedure that records gastric myoelectric activity (GMA) through cutaneous electrodes attached to the epigastrium. GMA is a record of gastric rhythms that originate from the interstitial cells of Cajal (the gastric pacemaker region). Historically, EGG was developed in 1921 and became more popular in 1990 [16]. Food ingestion stimulates stretch and mechanosensitive receptors in the stomach, which send vagal sensory signals to cortical and subcortical brain areas to produce the satiation perception. Reduced sensitivity to this sensory drive has been suggested to result in binge eating in patients with EDs [17]. The literature includes few studies measuring gastric neuromuscular activity and interoceptive signals in patients with EDs. van Dyck et al. [14] stated that patients with BED and BN have delayed satiety and abnormal GMA. Ogawa et al. [18] reported that the normal 3 cpm gastric activity in the ED group was lower than that of healthy controls, and the duration of the disease correlated with the percentage of bradygasteria. Diamanti et al. [19] found that BN produced abnormal GMA in adolescents, while anorexia nervosa did not. These reports suggest the role of GMA disturbances in the development and progression of binge eating and BN. In a previous pilot study, the relationship between GMA and body composition in adults with and without obesity was evident [20]. Disturbed GMA and abnormal satiation in patients with BED and BN may alter body composition in terms of the fat mass to fat-free mass ratio. Consequently, this altered body composition predisposes to various metabolic comorbidities, creating another challenge in the treatment of BED and BN. Accordingly, we hypothesize that women with EDs (specifically, BED or BN) may have an association between body composition and gastric myoelectric activity. To the best of our knowledge, this is the first study examining GMA in relation to body composition among patients with BED and BN. Measuring GMA might be a promising method to indicate the progress of BED and BN and may be linked to body composition changes associated with the disease. Accordingly, this study aimed to examine the myoelectrical activity of the stomach in women with BED and BN and the relationship between GMA and body composition in ED patients and age–BMI-matched controls.

## 2. Materials and Methods

### 2.1. Study Design, Participants, and Sample Size

**Figure 1:**
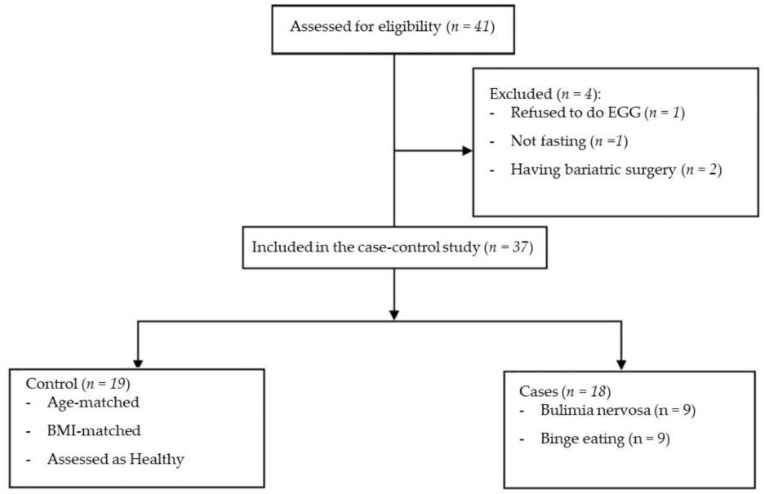
Flow diagram for recruitment of the participants for the matched case–control study.

This study was a matched case–control study conducted between February and December 2022 at the Clinical Nutrition Clinic, College of Applied Medical Sciences, and the Psychiatric Clinic, College of Medicine, King Saud University, Riyadh, KSA. A sample size of 38 participants (19 per group) was calculated using the G* power software (G*Power 3.1.9.7, Heinrich Heine University, Dusseldorf, Germany) to meet 80% power and an *α* level of 0.05 based on the previous finding of the GMA difference between ED patients and healthy controls [19]. Diamanti et al. [19] stated that the mean ± SD of postprandial gastric rhythm at 3 cycles per minute of the control group (n = 16) was 83.9 ± 17.7%, and for the bulimic group (n = 10), it was 45.2 ± 29.6% (effect size of 1.587) resulting in a minimum sample size of 16 (8 per group). Recruitment was conducted via an open invitation for participation which was announced via social media and hospital fliers. The participants included in this study were aged 18 to 60 years, had no gastrointestinal (GIT) diseases or previous surgeries, and were not taking medications affecting GIT functions (such as prokinetic, anti-emetic, and non-steroidal anti-inflammatory medications) or medications affecting hydration status or metabolic rate. In addition, they had no history of existing chronic diseases, such as cancer or heart disease. Pregnant women or those with other psychiatric disorders were also excluded. The ED group consisted of patients meeting the fifth edition of the Diagnostic and Statistical Manual of Mental Disorders (DSM-5) criteria for BED or BN, while age- and BMI-matched women without an ED were selected as the healthy controls (Figure 1). All participants were screened for the presence of EDs using the Eating Disorder Examination Questionnaire, version 6 (EDE-Q 6.0) [12]. The number of binge episodes was assessed by asking the participants how many binge eating episodes per week they had in the previous 3 months, in addition to using the EDE-Q 6.0 questionnaire. Informed consent was given; the participants were instructed about the study purpose, setting, procedure, and benefits, and their participation was voluntary. The Institutional Review Board (IRB) of the College of Medicine, King Saud University, approved the study protocol under reference number 22/0056/IRB, dated 18 January 2022. 

### 2.2. Demographic Data

Self-reported demographic data were obtained, including age, marital status, education status, and income level. According to the General Authority for Statistics in Saudi Arabia (GASTAT), Saudi workers’ monthly average wage in four sectors was SAR 10,238 (about 123 k SAR/year). Therefore, the annual income of 120 k SAR/year was considered the average [21]. Based on this, we divided the sample into three levels: <120 k as the low-income class, 120–180 k as the middle-income class, and >180 k as the high-income class. 

### 2.3. Anthropometric Measurement

Weight (kg) and height (cm) were measured manually using a Seca scale (Seca Co., Hamburg, Germany). BMI was calculated by dividing the weight by the square of the height (kg/m^2^). The participants were matched based on their BMI. The waist circumference (WC) and hip circumference (HC) were measured twice, and the average of the two measurements was considered for analysis.

### 2.4. Body Composition Analysis

Body fat percentage (BFP), fat mass (FM), fat free-mass (FFM), visceral fat rating (VFR), muscle mass (MM), and total body water (TBW) were measured using segmental multifrequency bioelectrical impedance (TANITA BC-418, Tanita Co., Tokyo, Japan). All measurements were performed in the morning (8:00 to 11:30 a.m.) for both groups. 

### 2.5. Measuring the GMA

The GMA was measured via multichannel electrogastrography (EGG) with a water load test (3CPM Company, Sparks, MD, USA). The EGG electrodes were placed on the skin at three sites: (a) mid-clavicular line, approximately two inches below the left costochondral margin; (b) mid-clavicular line on the right side, two inches below the right costochondral margin; (c) midline between the xiphoid and umbilicus. The EGG procedures are detailed in previous studies [16,22].

During the test, the participants were positioned in a comfortable supine position in complete rest in a room with low lighting. The pre-prandial EGG recording was conducted for 10 min; then, the participant was asked to drink plain water until satiety. The EGG recording continued for another 30 min. The EGG recording was divided into four periods for analysis: (a) baseline; (b) 10 min postprandial (10 min), from 0 to 10 min after water ingestion; (c) 20 min, from 10 to 20 min; (d) 30 min, from 20 to 30 min. The parameters used for analysis were the volume of water loading, percentage of power in the 0 to 15 CPM frequency range, and average dominant frequency (ADF) in each period. The distribution of average power by frequency region included bradygasteria (BradyG, 1.0–2.5 CPM), normogastria (NormoG, 2.5–3.75 CPM), tachygastria (TachyG, 3.75–10 CPM), and duodenal rhythm (Duodenal, 10–15 CPM).

### 2.6. Statistical Analysis

The study parameters are presented as means ± SDs or percentages. The Shapiro–Wilk test was used to test the normality. The Fisher’s Exact test was used for categorical variables. For continuous variables, the Mann–Whitney U test was used to compare the results among the study groups (control and ED) in addition to the comparison of the BMI < median and BMI ≥ median subgroups. The Kruskal–Wallis test was used to compare the results among the three groups (control, BED, and BN). A significant Kruskal–Wallis test was followed by a Mann–Whitney test to examine the multiple intergroup differences using Bonferroni corrections. Additionally, the Spearman correlation was used to investigate the correlation of GMA with body composition parameters. All differences were considered significant if the *p*-value was <0.05. The statistical analysis was performed using the Statistical Package for the Social Sciences (SPSS, version 29, Chicago, IL, USA).

## 3. Results

### 3.1. Demographic Results

Thirty-seven women were included in this study, comprising eighteen subjects with an ED (BED = 9 and BN = 9) and nineteen matched control subjects. The mean ± SD of the control and ED group participants’ age was 23.32 ± 3.01 and 23.89 ± 4.29 years, respectively. The number of binges in the BED subgroup was 2.89 ± 1.05 times, while in the BN subgroup, it was 2.44 ± 1.24 times. The mean ± SD of purge episodes in the BN subgroup was 4.78 ± 3.36. The income level was significantly higher among the ED patients compared to the controls (*p* = 0.022). However, the groups had no significant differences in academic, marital status, and sleeping hours. In addition, the percentage of participants with depression and anxiety was significantly higher in ED patients (27.8%) than in the controls (0.0%) (Table 1).

### 3.2. Body Composition between Study Groups

Table 2 presents the body composition measurements between subjects with ED and the BMI-matched controls. There were no statistically significant differences in body composition between the control group (n = 19) and the ED group (n = 18). Subdividing the ED group into BED and BN subgroups showed differences compared with the control. The BED group had a higher BMI, WC, HC, BFP, and fat mass than the BN and control groups (*p* < 0.05). Regarding muscle mass, fat-free mass, total body water, and visceral fat rating, there were significant differences between the BN group and the controls. The comparison of the BN and BED groups showed that participants with BN had a significantly lower BMI, FM, HC, BFP, muscle mass, FFM, TBW, and VFR (Table 2). 

### 3.3. EGG Recordings among Subgroups

Compared to the controls, the water load volume was significantly higher in the patients with ED. The comparison of the ED subgroups showed that water load volume was significantly higher in the BN subgroup (but not the BED subgroup) compared to the control group. Compared to the control group, patients with ED showed a lower percentage of tachygastria times in the Min20 phase (23.3 ± 10.3 vs. 16.8 ± 8.3, *p*-value = 0.042). A comparison of the subgroups with the controls showed a significantly lower percentage of tachygastria times in the BED but not the BN subgroup. The mean of ADF in the Min40 phase in the ED group was 1.0 ± 0.83 versus 1.5 ± 0.85 for the controls, with the *p*-value near significance (0.060). The percentage of normogastria times at the Min10 phase was higher in the BN subgroup compared to the BED subgroup. However, no other significant differences in EGG parameters were detected (Table 3).

Further analysis of the effect of body mass index (BMI) on the EGG parameters in the ED and control groups is shown in Table 4. The sample in each group was divided into two subgroups based on the BMI median (BMI < Median and BMI ≥ Median subgroups). The BMI median in the control group was 26.8 kg/m^2^, and in the ED group, it was 27 kg/m^2^. None of the differences in parameters were statistically significant. 

### 3.4. Correlations of the EGG Parameters with Body Composition and Number of Binges 

There was a statistically significant inverse correlation between ADF-Min20 and BFP and fat mass (r = −0.700 and 0.683, respectively, *p* < 0.05) (Table 5). Other groups failed to show any significant correlations. Water load volume correlated positively with the number of binges in both the BED and BN subgroups (r = 0.810 and 0.797, respectively, *p* < 0.05) (Table 6).

## 4. Discussion

This study examined the GMA status in patients with an ED (specifically BED and BN) and age–BMI-matched controls. In addition, we tested the association between GMA and body composition. Compared to healthy controls, there was a higher percentage of depression and anxiety in the ED group. This is an important finding since depression can affect food intake and body composition due to the patients’ eating behaviors and their feelings of guilt after eating, which can lead to a vicious cycle [23]. Moreover, patients with EDs were more likely to have a high income. This was consistent with some previous reports that EDs are frequent in populations with high socio-economic and low social status [24]. Another opinion reported no association between EDs and income. However, other factors such as employment status, education, and urbanicity had some impact on the prevalence of EDs in the Australian population [24]. Women with a higher socio-economic class are more likely to be easily influenced by sociocultural body ideals. Frequent and incorrect dieting increases the risk of the development of EDs [24].

Patients with EDs ingested more water than the control group, indicating a significantly higher intragastric gastric volume in the patients with ED. This finding was consistent with a previous study conducted on BED and BN patients [14]. This phenomenon of high water intake in the ED group found in this present study may indicate that ED patients might override their satiety level, thus increasing the challenge to stop eating, as suggested by Sysko et al. [25]. Collectively, they reported that patients with BED might have satiety disturbances similar to those described in patients with BN [25]. Moreover, Geliebter et al. [26] found that patients with BED have a large gastric capacity as assessed via test meal maximum tolerated volume and an intragastric balloon. Patients with BN also showed similar large gastric volumes, even larger than that in patients with obesity [27]. This high gastric volume may be due to disturbances in secretory patterns of insulin and meal-related gastric hormones such as ghrelin [26]. This large WL volume was not due to changes in BMI. Table 4 shows that there were insignificant differences between patients with ED who had a BMI greater than the median and those with a BMI less than the median. Previous reports from our research group indicated that patients with severe obesity have higher WL volumes than health controls, while patients with overweight and those with non-morbid obesity had insignificant differences regarding the WL volume [22]. The BMI range of the participants in this current study was in the overweight to non-severe obesity range. Moreover, Riezzo et al. [28] reported that BMI, age, and gender do not affect GMA.

This study showed a lower percentage of post-prandial tachygastria times in the ED group. Compared to the matched controls, the BED subgroup (but not the BN subgroup) showed a lower percentage of tachygastria. Moreover, the percentage of normogastria at Min10 in the BED subgroup was lower than that of the control, while the opposite results were found for the BN subgroup. Moreover, the ADF was inversely correlated with FM and BFP in the BN but not the BED subgroup. Binge eating has been hypothesized to cause slow gastric emptying by hindering the neuropeptide cholecystokinin (CCK), which is responsible for satiety [26]. Moreover, it was reported that the percentages of normogastria were significantly lower in the patients with BN and BED compared to the control group, the power in the bradygastria range was higher in patients with ED, and GMA was negatively associated with the number of binge-eating episodes per week. Koch et al. [29] found that the 1–2 cpm range (bradygastria) was significantly higher in the patients with BN before and after the water loading than in the control group. However, this current study showed insignificant differences in bradygasteria times between patients with ED and BMI-matched controls. 

The use of BIA in the assessment of the body composition of patients with eating disorders was proved to be efficacious. The assessment of only weight changes during the diagnosis and treatment period does not accurately reflect the real changes in body composition [30]. Sophisticated body composition assessment techniques may not be available in clinical settings [31]. This current study used BMI-matched controls, so there was no significant difference in body composition between the control and ED groups. However, on subdividing the ED group into BED and BN subgroups, the comparison with the normal control showed another picture. The BED subgroup had a higher BMI, WC, HC, BFP, and fat mass than the control group. Moreover, the BN subgroup had a significantly lower BMI, WC, HC, BFP, and fat mass than the control and BED groups. Patients with BN usually use compensatory behaviors to prevent weight gain, such as self-induced vomiting, fasting, compulsive exercise, and laxative or diuretic use. However, BED patients do not. Indeed, a strong relationship between BED and weight gain has been reported in the literature [6,32]. Despite being lower than the normal control, the body composition parameters in the BN subgroup were still in the normal range. Normal BMI values have been reported in patients with BN in many previous studies [33,34]. On the other hand, patients with BED had an obesity-like body composition profile. This was consistent with previous reports that documented an overweight or obese status in patients with BED [35,36,37]. 

The interesting finding in this study was the negative correlation between fat mass and BFP with ADF. Abulmeaty et al. [38] studied the association between body composition in different obesity phenotypes and GMA and found that the metabolically obese normal weight group (which is the most similar group to those with BN) had a negative correlation between fat mass index (FM/square height in meters) and pre-prandial ADF. This may indicate a possible association between the accumulation of FM and the slowdown of GMA. Moreover, the water load volume was correlated with the number of binges in both the BED and BN subgroups, indicating that the greater the water load volume, the more severe the condition is. This finding was different from the findings of van Dyck et al. [14], which showed that the number of objective binge-eating episodes during the past 3 months was not correlated with the mean water load volumes or pre-prandial percentages of EGG power. 

Despite many strengths, this study has some limitations. The main limitation is the absence of a male sample. Initially, we started recruitment of both males and females to increase the ecological validity of the results. Nevertheless, only one male participant was willing to participate after a long period. This forced us to change our objective to concentrate only on women. Women are more interested in and seek treatment for eating disorders than men. Similarly, results on differences between the BED and BN subgroups need to be interpreted with some caution because of the small sample sizes in each subgroup. We did proper sample size calculations, and the sample size of the ED and matched control groups represent sufficient statistical power. However, the number in each subgroup was relatively small, mandating the use of nonparametric tests in the statistical analyses.

## 5. Conclusions

In conclusion, this GMA study showed higher water load volumes in the patients with EDs as well as in the BED and BN subgroups, which correlated with the disease severity denoted by the number of binges. Apart from the percentage of tachygastria times, the EGG power range was not significantly different among the study groups. In the BN group, ADF was associated with body adiposity. These findings may be a basis for further investigations to understand gastric disturbances in ED patients. 

## Figures and Tables

**Table 1:** Demographic characteristics of the study participants.

	Control (*n* = 19) n (%)	ED (*n* = 18) n (%)	*p* Value
Annual income level (SAR)			0.022
<120 k	7 (36.8)	1 (5.6)	
120–180 k	5 (26.4)	3 (16.7)	
>180 k	7 (36.8)	14 (77.7)	
Educational level			1.000
High school + diploma	4 (21.1)	3 (16.7)	
Bachelor	11 (57.8)	11 (61.1)	
Postgraduate	4 (21.1)	4 (22.2)	
Marital status			1.000
Single	17 (89.5)	17 (94.4)	
Married	2 (10.5)	1 (5.6)	
Sleeping hours per day			0.315
<5 h	1 (5.3)	2 (11.1)	
5–7 h	7 (36.8)	10 (55.6)	
>7 h	11 (57.9)	6 (33.3)	
Presence of depression and anxiety			0.020
Yes	0(0.0)	5 (27.8)	
No	19(100.0)	13 (72.2)	

Fisher’s Exact test.

**Table 2:** Body composition differences between ED patients and BMI-matched controls.

Variables	Control (*n* = 19) Mean (SD)	ED (*n* = 18) Mean (SD)			*p*-Value *	*p*-Value **
		ED (*n* = 18)	BED (*n* = 9)	BN (*n* = 9)		
BMI (kg/m²)	26.9 (5.8) ^a^	26.5 (6.9)	32.1 (4.7) ^b^	20.9 (3.2) ^c^	0.627	0.001
WC (cm)	78.3 (7.2) ^a^	76.7 (11.0)	85.2 (5.4) ^b^	68.2 (8.2) ^c^	0.939	0.001
HC (cm)	107.0 (8.7) ^a^	103.9 (17.9)	117.1 (11.6) ^b^	90.8 (12.6) ^c^	0.504	0.001
BFP	34.6 (8.0) ^a^	31.1 (13.9)	41.8 (6.8) ^b^	20.3 (10.3) ^c^	0.564	0.001
Muscle mass (kg)	40.8 (4.7) ^a^	40.9 (5.4)	44.6 (4.6) ^a^	37.2 (3.2) ^b^	0.927	0.009
Fat mass (kg)	24.7 (10.9) ^a^	23.6 (15.9)	36.3 (10.8) ^b^	10.9 (7.6) ^c^	0.738	0.001
Fat-free mass (kg)	43.0 (4.8) ^a^	42.9 (5.6)	47.0 (4.4) ^a^	38.8 (3.0) ^b^	0.738	0.001
Total body water (kg)	31.7 (3.6) ^a^	31.5 (4.1)	34.6 (3.1) ^a^	28.4 (2.2) ^b^	0.627	0.001
Visceral fat rating	4.5 (2.7) ^a^	4.7 (3.5)	7.1 (3.0) ^a^	2.3 (2.0) ^b^	0.988	0.005

cm, centimeter; kg, kilogram; HC, hip circumference; WC, waist circumference. BFP, body fat percent; IQR, interquartile range. * *p*-value calculated using the Mann–Whitney test examining the differences between the control and the ED (Total) groups. ** *p*-value calculated using the Kruskal–Wallis test was used to compare the results among the three groups (control, BED, and BN). Values with different superscripts are statistically significant.

**Table 3:** EGG patterns among study groups and subgroups.

Variables	Control (*n* = 19) Mean (SD)	ED (*n* = 18) Mean (SD)			*p*- Value *	*p*-Value **
		ED (*n* = 18)	BED (*n* = 9)	BN (*n* = 9)		
Water load	451.0 (135.3) ^a^	685.5 (328.2)	581.1 (311.5) ^a^	790.0 (327.7) ^b^	0.005	0.003
BL-BradyG	53.1 (17.0) ^a^	52.3 (14.3)	53.1 (14.3) ^a^	51.5 (15.1) ^a^	1.000	0.961
BL-NormoG	19.8 (11.4) ^a^	20.2 (9.9)	18.9 (9.7) ^a^	21.4 (10.6) ^a^	0.693	0.841
BL-TachyG	19.0 (8.9) ^a^	21.5 (9.1)	22.2 (9.6) ^a^	20.8 (9.1) ^a^	0.429	0.703
BL-Duodenal	7.9 (6.6) ^a^	5.8 (3.7)	5.6 (3.9) ^a^	6.0 (3.7) ^a^	0.447	0.710
Min10_BradyG	56.2 (18.1) ^a^	58.8 (15.1)	63.7 (16.3) ^a^	53.8 (12.6) ^a^	0.738	0.192
Min10_NormoG	15.7 (8.1) ^a^	16.3 (12.0)	10.8 (5.2) ^a^	21.9 (14.5) ^b^	0.627	0.059
Min10_TachyG	21.6 (11.2) ^a^	18.0 (11.0)	19.1 (12.3) ^a^	17.0 (10.1) ^a^	0.191	0.394
Min10_Duodenal	6.3 (6.6) ^a^	6.7 (7.4)	6.2 (6.8) ^a^	7.1 (8.4) ^a^	0.891	0.897
Min20_BradyG	53.6 (18.1) ^a^	54.2 (16.1)	55.7 (17.7) ^a^	52.7 (15.2) ^a^	0.855	0.965
Min20_NormoG	18.4 (9.6) ^a^	24.4 (15.3)	25.1 (14.6) ^a^	23.7 (16.9) ^a^	0.316	0.577
Min20_TachyG	23.3 (10.3) ^a^	16.8 (8.3)	16.3 (7.4) ^b^	17.3 (9.6) ^a^	0.042	0.287
Min20_Duodenal	5.5 (6.1) ^a^	4.4 (4.1)	2.7 (1.3) ^a^	6.1 (5.3) ^a^	0.727	0.613
Min30_BradyG	56.0 (18.1) ^a^	49.6 (20.2)	56.2 (16.1) ^a^	43.0 (22.6) ^a^	0.362	0.373
Min30_NormoG	18.2 (12.8) ^a^	22.8 (15.0)	2.1 (10.4) ^a^	24.4 (19.1) ^a^	0.316	0.604
Min30_TachyG	21.1 (10.0) ^a^	19.6 (11.3)	18.6 (9.5) ^a^	20.6 (13.3) ^a^	0.627	0.887
Min30_Duodenal	4.5 (3.9) ^a^	7.8 (14.0)	3.8 (1.9) ^a^	11.8 (19.5) ^a^	0.412	0.432
ADF-BL	1.8 (1.3) ^a^	1.6 (0.72)	1.6 (0.89) ^a^	1.5 (0.56) ^a^	0.659	0.907
ADF-Min10	2.0 (1.6) ^a^	1.8 (0.79)	1.5 (0.42) ^a^	2.0 (1.0) ^a^	0.704	0.788
ADF-Min20	1.6 (0.70) ^a^	1.6 (0.75)	1.6 (0.71) ^a^	1.5 (0.84) ^a^	0.773	0.906
ADF-Min30	1.5 (0.85) ^a^	1.0 (0.83)	1.1 (0.91) ^a^	0.92 (0.77) ^a^	0.060	0.149

BradyG, bradygastria; BL, baseline; NormoG, normogastria; TachyG, tachygastria; ADF, average dominant frequency. * *p*-value calculated using the Mann–Whitney test examining the differences between the control and the ED (total) groups. ** *p*-value calculated using the Kruskal–Wallis test to compare the results among the three groups (control, BED, and BN). Values with different superscripts are statistically significant.

**Table 4:** EGG patterns among study groups, stratified by body mass index (BMI).

Variables	Control (*n =* 19) Mean (SD)		*p*-Value	ED (*n* = 18) Mean (SD)		*p*-Value
	BMI < 26.8 (*n =* 9)	BMI ≥ 26.8 (*n =* 10)		BMI < 27 (*n =* 9)	BMI ≥ 27 (*n =* 9)	
Water load	480.0 (99.4)	425.0 (162.2)	0.447	744.4 (325.0)	626.7 (340.0)	0.297
BL-BradyG	50.0 (16.3)	56.0 (18.1)	0.243	51.5 (15.3)	53.2 (14.2)	0.931
BL-NormoG	23.8 (13.7)	16.3 (8.2)	0.156	19.9 (7.1)	20.5 (12.7)	0.796
BL-TachyG	18.0 (7.4)	20.0 (10.5)	0.968	22.7 (10.6)	20.4 (7.9)	0.730
BL-Duodenal	8.2 (4.9)	7.7 (8.2)	0.243	5.9 (3.8)	5.9 (3.9)	0.931
Min10_BradyG	58.5 (18.6)	54.3 (18.5)	0.780	54.8 (14.0)	62.8 (15.9)	0.161
Min10_NormoG	15.1 (10.6)	16.4 (5.5)	0.447	21.8 (14.7)	11.0 (5.2)	0.094
Min10_TachyG	20.6 (14.4)	22.5 (8.2)	0.356	16.3 (10.2)	19.9 (12.1)	0.436
Min10_Duodenal	5.8 (4.8)	6.7 (8.1)	0.968	7.1 (8.5)	6.3 (6.8)	0.796
Min20_BradyG	51.4 (24.1)	55.7 (11.5)	0.497	50.9 (15.5)	57.6 (16.9)	0.546
Min20_NormoG	18.2 (9.8)	18.8 (10.8)	0.968	25.9 (17.0)	23.00 (14.5)	0.666
Min20_TachyG	22.6 (13.1)	22.0 (10.5)	0.842	17.2 (9.5)	16.6 (7.6)	0.666
Min20_Duodenal	7.9 (7.0)	3.5 (4.7)	0.053	6.1 (5.4)	2.8 (1.4)	0.436
Min30_BradyG	56.2 (21.1)	56.0 (16.3)	0.968	40.5 (22.1)	58.9 (13.9)	0.094
Min30_NormoG	17.5 (13.9)	18.9 (12.5)	0.780	26.1 (19.2)	19.6 (9.5)	0.546
Min30_TachyG	20.1 (11.9)	22.2 (8.5)	0.549	21.6 (13.5)	17.7 (9.0)	0.489
Min30_Duodenal	6.3 (5.1)	2.9 (1.4)	0.095	11.8 (19.5)	3.9 (2.0)	0.222
ADF-BL	1.8 (0.73)	2.0 (1.76)	0.447	1.7 (0.91)	1.5 (0.52)	0.796
ADF-Min10	1.8 (0.74)	2.2 (2.21)	0.905	2.1 (1.1)	1.6 (0.44)	0.340
ADF-Min20	2.0 (0.90)	1.4 (0.31)	0.133	1.7 (0.84)	1.5 (0.71)	0.297
ADF-Min30	1.4 (1.02)	1.8 (0.68)	0.065	1.1 (0.98)	1.0 (0.70)	0.863

BradyG, bradygastria; BL, baseline; NormoG, normogastria; TachyG, tachygastria; ADF, average dominant frequency. *p*-value was calculated using the Mann–Whitney test examining the differences between the subgroups BMI < median and BMI ≥ median.

**Table 5:** Correlations between gastric myoelectrical activity and body composition among subgroups.

Variables	Control (*n* = 19) r (*p*-Value)				BED (*n* = 9) r (*p*-Value)				BN (*n* = 9) r (*p*-Value)			
	BFP	FM	FFM	TBW	BFP	FM	FFM	TBW	BFP	FM	FFM	TBW
Water load (mL)	0.227 (0.350)	0.189 (0.437)	0.076 (0.757)	0.002 (0.994)	0.142 (0.715)	−0.017 (0.966)	0.017 (0.966)	−0.017 (0.966)	0.332 (0.383)	0.264 (0.493)	0.579 (0.102)	0.579 (0.102)
ADF-BL	−0.118 (0.629)	−0.126 (0.609)	−0.134 (0.586)	−0.209 (0.391)	0.133 (0.732)	−0.150 (0.700)	−0.067 (0.865)	−0.300 (0.433)	0.383 (0.308)	0.400 (0.286)	−0.017 (0.966)	−0.017 (0.966)
ADF-Min10	−0.098 (0.689)	−0.156 (0.523)	−0.376 (0.112)	−0.376 (0.113)	0.067 (0.865)	−0.167 (0.668)	0.117 (0.765)	0.350 (0.356)	0.067 (0.865)	0.083 (0.831)	0.033 (0.932)	0.033 (0.932)
ADF-Min20	−0.288 (0.232)	−0.320 (0.181)	0.072 (0.772)	−0.008 (0.974)	−0.100 (0.798)	0.200 (0.606)	0.200 (0.606)	0.433 (0.244)	−0.700 (0.036) *	−0.683 (0.042) *	0.100 (0.798)	0.100 (0.798)
ADF-Min30	0.353 (0.139)	0.364 0.125)	0.417 (0.076)	0.404 (0.086)	0.109 (0.780)	−0.092 (0.813)	0.092 (0.813)	−0.101 (0.796)	−0.203 (0.600)	−0.254 (0.509)	0.136 (0.728)	0.136 (0.728)

ADF, average dominant frequency; BradyG, bradygastria; BL, baseline; NormoG, normogastria; TachyG, tachygastria; Min, minute; FFM, fat-free mass; TWB, total body water; r, Spearman correlation coefficient. * means statistically significant.

**Table 6:** Correlations between gastric myoelectrical activity and number of binges among subgroups.

Variables	ED (*n* = 18) r (*p*-Value)	BED (*n* = 9) r (*p*-Value)	BN (*n* = 9) r (*p*-Value)
Water load (mL)	0.538 (0.021)	0.810 (0.008)	0.797 (0.010)
ADF-BL	0.204 (0.417)	0.576 (0.104)	−0.139 (0.722)
ADF-Min10	0.018 (0.942)	0.151 (0.699)	−0.069 (0.859)
ADF-Min20	0.105 (0.679)	−0.053 (0.892)	0.173 (0.656)
ADF-Min30	0.237 (0.344)	−0.152 (0.696)	0.511 (0.160)

## Data Availability

Original data supporting these results are available on reasonable request from the corresponding author.
